# Clinical Efficacy of Bladder Neck Injection of Botulinum Toxin A in Treating Neurogenic and Non-Neurogenic Voiding Dysfunctions Due to Bladder Neck Dysfunction

**DOI:** 10.3390/toxins17060289

**Published:** 2025-06-06

**Authors:** Yu-Shuang Lee, Yu-Khun Lee, Tien-Lin Chang, Cheng-Ling Lee, Sheng-Fu Chen, Jia-Fong Jhang, Yuan-Hong Jiang, Hann-Chorng Kuo

**Affiliations:** 1 Department of Urology, Hualien Tzu Chi Hospital, Buddhist Tzu Chi Medical Foundation, Hualien 970, Taiwan; n10235651@gmail.com (Y.-S.L.); zxc13912@gmail.com (T.-L.C.); 2 Department of Urology, Hualien Tzu Chi Hospital, Buddhist Tzu Chi Medical Foundation, Tzu Chi University, Hualien 970, Taiwan; leeyukhun@gmail.com (Y.-K.L.); leecl@hotmail.com (C.-L.L.); madaux@yahoo.com.tw (S.-F.C.); alur1984@hotmail.com (J.-F.J.); redeemer1019@yahoo.com.tw (Y.-H.J.)

**Keywords:** bladder neck dysfunction (BND), botulinum toxin A (BoNT-A), neurogenic bladder

## Abstract

Bladder neck dysfunction (BND) is a pathophysiology associated with voiding dysfunction in patients with neurogenic or non-neurogenic voiding dysfunction. Botulinum toxin A (BoNT-A) injection is a minimally invasive alternative for treating bladder outlet dysfunction; however, its efficacy for BND has not been well established. In this retrospective study, 41 patients with videourodynamic study-confirmed BND who failed medical therapy received a transurethral bladder neck injection of 100-U BoNT-A. Treatment outcomes were assessed using the Global Response Assessment. After BoNT-A injection, the patients were followed up and subsequent urological management was recorded. At 6 months, 65.9% of the patients reported satisfactory outcomes (26.8% successful and 39.0% improved). Patients with non-neurogenic BND had the highest satisfaction rate, higher than those with neurogenic BND (NBND) with and without detrusor sphincter dyssynergia (DSD). Among patients without detrusor acontractility (DA), a higher bladder outlet obstruction index predicted treatment failure. Patients with pure BND confirmed by urodynamics may benefit more from BoNT-A injections, whereas those with high baseline voiding detrusor pressure or spinal cord injury with detrusor sphincter dyssynergia may have less favorable results. Bladder neck BoNT-A injections for treating BND-associated voiding dysfunction did not achieve very successful outcomes. Only 26.8% of the patients had successful treatment outcomes, while 39.0% had improved outcomes and 34.1% failed the treatment.

## 1. Introduction

Voiding dysfunction is common in patients with neurogenic and non-neurogenic lower urinary tract dysfunctions (LUTDs). The bladder neck plays an important role in patients with neurogenic LUTDs, bladder outlet obstruction (BOO), or detrusor underactivity (DU) [1,2,3]. A hyperactive or contractured bladder neck during voiding obstructs the bladder outlet and causes incomplete voiding and detrusor overactivity (DO) [4]. Patients with bladder neck dysfunction (BND) may experience ineffective detrusor contractility due to autonomic nervous dysregulation, thereby causing incomplete bladder emptying [3]. Patients with DU due to peripheral nerve neuropathy or chronic urinary retention may not be able to void by abdominal straining due to a nonrelaxing bladder neck [5]. Patients with high-level spinal cord lesions may develop autonomic dysreflexia and bladder neck obstruction during reflex urination, resulting in high voiding pressure and upper urinary tract deterioration [6,7].

Identification of BND or bladder neck obstruction may help decide the treatment strategy and precisely improve voiding through effective management. Currently, BND can be diagnosed by conducting a videourodynamic study (VUDS) [8]. Cystourethrographic images of voiding that demonstrate a non-opening bladder neck with a high or normal voiding detrusor pressure can help accurately diagnose BND [9]. After a precision diagnosis, an alpha-adrenoceptor blocker can be administered to relax the bladder neck [10,11]. For patients with unsatisfactory treatment outcomes, transurethral incision of the bladder neck (TUI-BN) can be recommended to rapidly restore efficient voiding and improve voiding symptoms [9]. However, patients with BND may not accept the proposal of surgical treatment. In such cases, botulinum toxin A (BoNT-A) injection into the bladder neck may be a minimally invasive alternative for treating BND.

For 3 decades, BoNT-A injections have been widely used to treat neurogenic or non-neurogenic DO refractory to antimuscarinic therapy [12]. Patients with neurogenic detrusor sphincter dyssynergia (DSD), non-neurogenic dysfunctional voiding (DV), or DU with an open bladder neck have also been treated with urethral sphincter BoNT-A injection [13,14,15]. Although only approximately half of the patients treated with BoNT-A injection into the urethral sphincter had satisfactory results, most doctors recommend this minimally invasive therapy as the first-line treatment to patients who have failed medical treatment and are afraid of surgery [13]. However, clinical research on the efficacy of BoNT-A injection in the treatment of BND is limited. Therefore, this study retrospectively analyzed the treatment outcomes of BoNT-A injection in NBND or non-neurogenic BND and attempted to identify predictive factors for satisfactory and failed treatment outcomes.

## 2. Results

In total, this study included 41 patients (33 men and eight women) who had VUDS-proven BND received BoNT-A injection (100 U) into the bladder neck for voiding dysfunction. The mean age was 43.6 ± 17.1 years (range, 4–74 years). Among the patients, 34 (82.9%) had normal detrusor contractility and 7 (17.1%) had DA. There was no adverse event recorded in this retrospective analysis. Six months after the BoNT-A injection, 11 (26.8%) patients had successful treatment outcomes, 16 (39.0%) had improved outcomes, and 14 (34.1%) failed the treatment. The overall rate of satisfactory results was 65.9% at the 6-month follow-up.

Table 1 presents the baseline VUDS parameters between patients with satisfactory treatment outcomes and those with treatment failure. According to the VUDS findings, 11 patients had BND, 13 had BND plus DV, 3 had NBND, and 14 had NBND plus DSD. Voiding Pdet was <20 cmH_2_O in 14 patients, 20–40 cmH_2_O in 13 patients, and ≥40 cmH_2_O in 14 patients. No significant differences in treatment outcomes were observed among patients with different VUDS findings or different detrusor pressure subgroups. Analysis of the baseline VUDS parameters revealed that patients with BND had the highest rate of satisfactory outcomes, while those with NBND or NBND plus DSD had lower rates. However, the difference did not reach statistical significance. The baseline VUDS parameters did not predict treatment outcomes. Patients with treatment failure tended to be older and had a higher BOO index than those with satisfactory outcomes, although the differences were not statistically significant.

To further investigate outcome predictors, patients were stratified into those with normal detrusor contractility (n = 34) and those with DA (n = 7). In the subgroup of patients without DA, the BOO index was found to be significantly higher in the treatment failure group than in those with improved or successful outcomes (Table 2). Analysis of the baseline VUDS parameters revealed that patients with BND had the highest satisfactory rates, while those with NBND or NBND plus DSD had lower rates. However, the difference did not reach a statistical significance in overall patients or in patients without DA. Patients without DA and treatment failure had a higher BOO index than those with satisfactory treatment outcomes (52.5 ± 30.8 vs. 27.7 ± 23.8; *p* = 0.047), indicating that patients with greater bladder outlet resistance have less favorable treatment outcomes to BoNT-A injections into the bladder neck.

Among the 41 patients, 26 received concurrent BoNT-A injections into the urethral sphincter during the same session as the bladder neck injection to manage DV, DSD, or a nonrelaxing urethral sphincter and to improve VE. Among these 26 patients, 18 (69.2%) had improved or successful outcomes and 8 (30.8%) had treatment failure (*p* = 0.71).

We also attempted to identify predictive factors for treatment failure following BoNT-A injection. Due to the small sample size, we conducted a univariate analysis to screen potential predictors. Only age had a *p*-value < 0.2. Therefore, subsequent multivariate logistic regression could not be performed.

**Figure 1:**
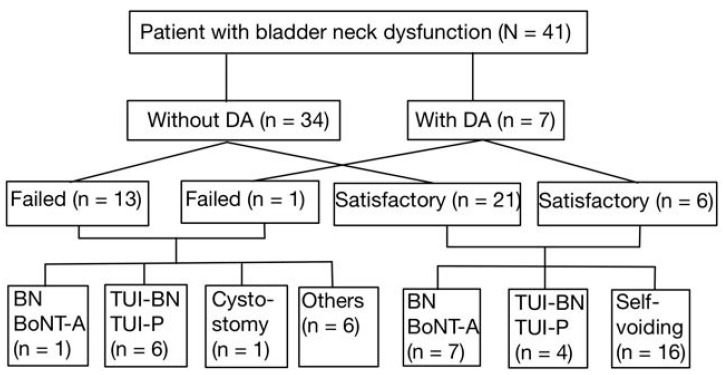
The patient flow and the subsets who received additional interventions.

After the first BoNT-A injection into the bladder neck, 7 out of 27 patients (25.9%) who had a satisfactory outcome underwent repeat BoNT-A injections following waning of the initial treatment response. In addition, 4 of these 27 patients (14.8%) received TUI-BN or TUI-P to achieve more permanent therapeutic results. The other 16 patients voided smoothly during the follow-up period. Among the 14 patients who failed the first BoNT-A injection into the bladder neck, one (7.1%) received a repeat BoNT-A injection, six (42.9%) underwent TUI-BN or TUI-P to improve VE, and one (7.1%) underwent suprapubic cystostomy for the permanent management of NBND plus DSD. All patients who underwent TUI-BN or TUI-P had satisfactory treatment outcomes in the long-term follow-up (Figure 1).

## 3. Discussion

This study is a retrospective case series and exploratory observational study aimed at evaluating the efficacy of bladder neck BoNT-A injection for treating VUDS-confirmed BND or NBND. The findings indicate that patients with non-neurogenic BND achieved the highest satisfactory rate, whereas those with NBND or NBND plus DSD exhibited lower response rates. Moreover, treatment failure was associated with a significantly higher BOO index in patients without DA. These results suggest that although BND was evident in VUDS, other urethral dysfunctions such as DV and DSD coexist with BND and result in failure of BoNT-A injection into the bladder neck. Due to the small sample size and exploratory nature of this study, only univariate analyses were conducted. No statistically significant predictors for treatment failure were identified, and multivariate regression analysis was not feasible.

BND can be divided into high-pressure and low-pressure subtypes [8]. High-pressure BND is usually accompanied by an anatomical obstruction, such as stenosis or fibrosis of the bladder neck, which prevents the bladder neck from opening properly during voiding, thereby increasing the pressure within the bladder to maintain urination [4,16]. In contrast, low-pressure BND results from detrusor muscle weakness and insufficient intravesical pressure to void the bladder [16]. BND is frequently associated with DV or urethral sphincter dysfunction in men and women. DV occurs when the external urethral sphincter fails to relax properly during voiding and may even paradoxically contract, thereby obstructing urinary flow [17]. Similarly, in patients with spinal cord lesions and DSD, the detrusor contracts against a closed sphincter, leading to high voiding pressure and inefficient bladder emptying [18].

BoNT-A injection into the bladder neck inhibits acetylcholine release at neuromuscular junctions, thereby inducing transient chemical denervation of the bladder neck smooth muscle [19]. However, in patients with concomitant DV or DSD, the primary dysfunction lies within the urethral smooth muscle or external sphincter rather than the bladder neck itself. BoNT-A injection into the bladder neck alone may not sufficiently improve VE. This may explain the moderate overall satisfaction rate (65.9%) observed at 6 months. Similarly, this pathophysiology of voiding dysfunction can explain why the non-neurogenic BND group had the highest satisfactory rate, whereas those with NBND had lower satisfactory rates, suggesting that patients with non-neurogenic BND without DV represent pure BND.

According to the location of the neurological injury, NBND can be classified into different subtypes, each of which exhibits distinct pathophysiological characteristics. Upper motor neuron lesions are caused by damage above the pontine micturition center. The VUDS characteristic is DO; however, the coordination between the detrusor and external sphincter is maintained, thereby preventing high bladder pressure [20]. Lesions between the pontine micturition center and sacral spinal cord are commonly observed in the cervical and thoracic spinal cord. The primary feature of lower urinary tract dysfunction is DSD, where the detrusor contracts simultaneously with the involuntary contraction of the external urethral sphincter, leading to increased resistance to urination, elevated bladder pressure, and potential bladder–ureteral reflux, which may result in upper urinary tract damage [21]. Sacral spinal cord or peripheral nerve injury may lead to detrusor muscle weakness or dysfunction of the external urethral sphincter. Patients often present with urination difficulty, urinary retention, and even overflow incontinence [20]. In previous studies, BoNT-A injection into the detrusor muscle effectively reduced the occurrence of autonomic dysreflexia (AD). This may be due to the decrease in detrusor muscle pressure and the increase in bladder compliance, which subsequently reduces the triggers for AD [22,23]. The impact of BoNT-A injection into the urethral sphincter on AD is not as significant as that of intravesical BoNT-A injection; however, it still provides a certain degree of improvement in AD [23]. BoNT-A injection into the bladder neck may reduce AD but is not adequate for the resolution of DSD; therefore, VE was not effectively improved by BoNT-A injection [24].

Analysis of the baseline characteristics of patients with normal detrusor contractility revealed that patients who experienced treatment failure had a significantly higher BOO index, suggesting that increased bladder outlet resistance is a critical factor limiting the effectiveness of BoNT-A injection for managing BND. Patients with BND may have anatomical or functional BOO, resulting in different voiding pressure, low Qmax, or PVR. BoNT-A injection into the bladder neck can reduce smooth muscle tone to facilitate spontaneous voiding at a lower voiding detrusor pressure. However, 100 U of BoNT-A may not be adequate for all patients with BND, particularly those with a fibrotic bladder neck or NBND [25]. This is also the reason why all patients who failed the initial BoNT-A injection into the bladder neck can achieve successful treatment outcomes after TUI-BN or TUI-P. Because BoNT-A injection is a minimally invasive therapy, it can still be a treatment of choice in patients with VUDS-identified BND.

The pathophysiology of non-neurogenic BND is complex. A tight bladder neck during voiding may be primarily due to increased sympathetic tone [26] or secondarily due to detrusor underactivity or DA [27]. Furthermore, the bladder neck is a continuation of the trigonal and urethral smooth muscles, and BND is often combined with DV or urethral dysfunction [16]. Patients with VUDS-confirmed BND may present with dysfunctional voiding after TUI-BN and may continue to suffer from voiding dysfunction due to DV [3]. Therefore, although BoNT-A injection is a minimally invasive option for managing BND, its efficacy remains limited in certain patient populations. Patients who did not achieve satisfactory treatment outcomes often required subsequent surgical intervention, such as TUI-BN or TUI-P, which resulted in significant improvement in voiding function [28]. These findings emphasize that although BoNT-A therapy may be an appropriate first-line option for select patients, surgical management remains the definitive treatment for those with persistent voiding dysfunction or inadequate response to injection therapy.

This study has several limitations that should be acknowledged. The small sample size may have limited the statistical power of certain analyses, contributing to the marginal significance observed for some predictive factors. Furthermore, as a retrospective analysis, potential selection bias cannot be excluded. Future studies with larger sample sizes are required to validate these findings and refine patient selection criteria for BoNT-A therapy.

## 4. Conclusions

This study demonstrated that BoNT-A injection into the bladder neck for the treatment of BND-associated voiding dysfunction does not achieve very successful outcomes. Only 26.8% of the patients had successful treatment outcomes, while 39.0% had improved outcomes and 34.1% experienced treatment failure. However, patients with VUDS-proven BND may achieve good therapeutic results and patients with a high Pdet at baseline and those with spinal cord injury and DSD may have less favorable outcomes after BoNT-A injection into the bladder neck.

## 5. Materials and Methods

### 5.1. Study Design and Setting

This was a retrospective case series and exploratory observational study conducted at the Department of Urology, Hualien Tzu Chi Hospital, Buddhist Tzu Chi Medical Foundation, Hualien City, Taiwan. This study was approved by the Institutional Review Board (IRB) of the Buddhist Tzu Chi Medical Foundation (IRB code 114-057-B, dated 19 March 2025). The requirement for informed consent was waived due to the retrospective nature of the study. All procedures were performed in accordance with the Declaration of Helsinki.

### 5.2. Participants

We retrospectively reviewed medical records from June 2005 to July 2023. Patients were eligible for inclusion if they were diagnosed with BND or NBND by VUDS and underwent at least one transurethral injection of BoNT-A into the bladder neck at our institution. Patients were excluded only if they had incomplete medical records, missing baseline VUDS data, or missing 6-month outcome assessment. A total of 41 patients met the inclusion criteria and were enrolled. The 41 patients were further divided into 7 with DA and 34 with normal detrusor contractility for subgroup analysis.

### 5.3. Baseline Evaluation and Diagnosis

All patients presented with voiding dysfunction, such as difficulty initiating or maintaining urination, often accompanied by abdominal straining and elevated postvoid residual (PVR) volume. All had been treated with alpha-adrenoceptor blockers with or without Baclofen for at least 3 months without symptom improvement. Baseline VUDS was performed according to the International Continence Society guidelines using a multichannel urodynamic system (Life-Tech Inc., Stafford, TX, USA) and a C-arm fluoroscope (Toshiba, Tokyo, Japan) [29]. Each patient underwent at least two reproducible pressure flow studies.

Parameters recorded included first sensation of filling (FSF), full sensation (FS), cystometric bladder capacity (CBC), bladder compliance, maximum flow rate (Qmax), detrusor pressure at Qmax (Pdet), PVR, sphincter electromyography (EMG) activity, voided volume, bladder outlet obstruction index (BOOI, defined as Pdet − [2 × Qmax]), bladder contractility index (BCI, defined as BCI = Pdet + 5 Qmax), and voiding efficiency (VE, defined as voided volume/bladder capacity × 100%). Voiding cystourethrography was performed with the C-arm positioned 30–45° from the buttocks to visualize the bladder neck, urethral sphincter, and distal urethra.

**Figure 2:**
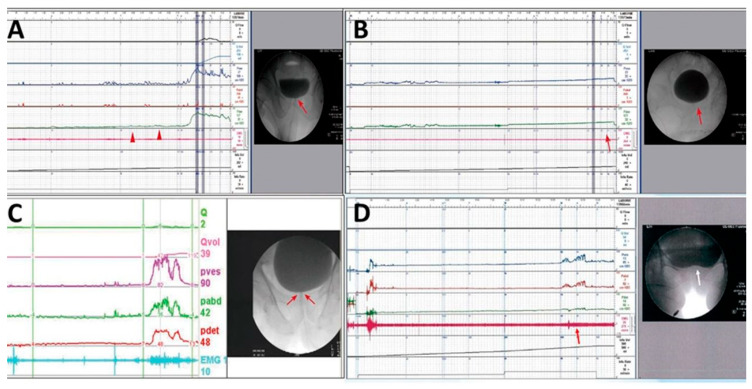
Different videourodynamic tracings of patients with non-neurogenic and neurogenic bladder neck dysfunction (BND). (**A**) A male patient with high voiding pressure BND (red arrow); (**B**) a male patient with detrusor underactivity and BND (red arrows); (**C**) a female patient with spinal cord injury, high voiding pressure, BND (red arrows), and detrusor sphincter dyssynergia; and (**D**) a female patient with low detrusor contractility, BND (white arrow), and dysfunctional voiding (red arrow).

Patients with severe difficulty in urination, straining while voiding, large PVR, and a narrow bladder neck observed during voiding in VUDS were diagnosed with BND (Figure 2A). Patients with a voiding Pdet of <10 cmH_2_O, very low Qmax, and PVR of >150 mL were considered as having DU, whereas those with no detrusor contractility, straining while voiding, and PVR of >150 mL were considered as having detrusor acontractility (DA) (Figure 2B). Patients with chronic spinal cord injury who had a tight urethral sphincter with increased urethral sphincter EMG activity were diagnosed with DSD (Figure 2C). Patients with a narrow bladder neck with a narrow urethral sphincter during voiding in cystourethrography were considered to have BND and DV (Figure 2D).

### 5.4. Intervention

After informing the patients of the postoperative adverse events and potential complications of anesthesia and surgery, each patient was admitted for injection of BoNT-A into the bladder neck. Baseline VUDS was conducted within 2 weeks before the procedure. When patients were found to have DV or DSD, 100 U of BoNT-A was injected into the urethral sphincter along with the bladder neck BoNT-A injection. BoNT-A injections into the urethral sphincter were performed as previously described [30]. BoNT-A injections into the bladder neck were performed under intravenous general anesthesia using injection cystoscopy (Richard–Wolf, Knittingel, Germany) and a 23-G injecting needle. A total of 100 U of BoNT-A (onabotulinumtoxinA; Allergan, Irvine, CA, USA) dissolved in 5 mL of normal saline was injected at five sites of the bladder neck. The injection sites were located at the 1, 3, 5, 7, and 9 o’clock positions of the bladder neck, approximately 5 mm in depth. One milliliter of the BoNT-A solution (20 U) was injected into each site. After the bladder neck and urethral sphincter injections, a 14 Fr Foley catheter was placed overnight and the patients were discharged the next morning. All BoNT-A injections were performed under the supervision of a single senior urologist (HCK) to reduce inter-operator variation. The operative time was under 20 min in all cases. Adverse events occurred with BN BoNT-A injection were treated and recorded if any.

### 5.5. Follow-Up and Outcome Assessment

Follow-up visits occurred approximately one week post-injection and continued for up to six months. The primary outcome was the self-reported Global Response Assessment (GRA) score at 6 months post-injection (scored from −3 (worst) to +3 (excellent)) for improvement of VE and difficulty [31]. The patients were also followed up for their Qmax and PVR after BoNT-A injections. If the patient had GRA improved by +1, this was considered an improved treatment outcome; +2 was a successful outcome and +3 was an excellent outcome, if the Qmax also improved and the PVR volume decreased. Patients with improved, successful, or excellent outcomes were considered to have satisfactory BoNT-A injection results. Patients with a GRA of ≤0 were considered failure to treatment. Patients who received further treatment for persistent voiding dysfunction were recorded and analyzed.

All patients were regularly followed up at the urology clinic. Repeat BoNT-A injections into the bladder neck or urethral sphincter were recommended if the initial treatment effect had disappeared. Patients were also recommended to undergo TUI-BN or transurethral incision of the prostate (TUI-P) if the symptoms of difficult urination did not improve or the improvement did not reach their expectations. The treatment outcomes of TUI-BN and TUI-P were also recorded.

### 5.6. Statistical Analysis

Statistical analyses were performed using Statistical Package for the Social Sciences version 16.0 (IBM Corp., Armonk, NY, USA). Continuous variables were presented as mean ± standard deviation (SD) and categorical data as numbers and percentages (%). Group comparisons were performed using the Fisher’s exact test for categorical variables and the Wilcoxon rank-sum test for continuous variables. All assessments were two-sided, and *p*-values < 0.05 were used to denote statistical significance. For predictive analysis of treatment failure, 7 patients with DA were excluded, leaving 34 patients for univariate analysis. Multivariable analysis was not performed due to the limited sample size. Given the exploratory nature of this study, the hypothesis-generating rather than confirmatory analysis was emphasized.

## Figures and Tables

**Table 1:** Baseline videourodynamic parameters between all patients with successful or improved outcomes and those with treatment failure.

Parameters	Satisfactory (n = 27)	Failed (n = 14)	*p*-Value
**Age (years)**	40.1 ± 18.1	50.4 ± 12.7	0.065
**VUDS parameters**			
**BND**	9 (33.3%)	2 (14.3%)	0.637
**BND + DV**	8 (29.6%)	5 (35.7%)	
**NBND**	2 (7.4%)	1 (7.1%)	
**NBND + DSD**	8 (29.6%)	6 (42.9%)	
**FSF (mL)**	144.5 ± 72.9	143.6 ± 56.1	0.970
**FS (mL)**	233.0 ± 103.9	217.3 ± 88.4	0.633
**US (mL)**	287.2 ± 123.7	268.5 ± 120.0	0.646
**Compliance**	70.7 ± 59.7	81.7 ± 96.7	0.655
**Pdet (cmH_2_O)**	33.3 ± 23.8	36.1 ± 30.4	0.746
**Qmax (mL/s)**	6.6 ± 5.2	8.0 ± 6.5	0.443
**Volume (mL)**	178.0 ± 175.0	170.6 ± 162.8	0.895
**PVR (mL)**	153.3 ± 156.2	171.4 ± 225.9	0.765
**CBC (mL)**	331.4 ± 142.1	342.0 ± 185.4	0.839
**VE**	0.52 ± 0.41	0.56 ± 0.41	0.807
**BOOI**	25.9 ± 22.8	41.4 ± 33.5	0.154
**BCI**	66.0 ± 36.2	76.1 ± 39.8	0.421

Abbreviations: BND: bladder neck dysfunction, NBND: neurogenic bladder neck dysfunction, DV: dysfunctional voiding, DSD: detrusor sphincter dyssynergia, FSF: first sensation of filling, FS: fullness sensation, US: urge sensation, Pdet: detrusor pressure, Qmax: maximum flow rate, PVR: post-void residual, CBC: cystometric bladder capacity, VE: voiding efficiency, BOOI: bladder outlet obstruction index, BCI: bladder contractility index.

**Table 2:** Baseline videourodynamic parameters of patients without DA and successful or improved treatment outcomes and those with treatment failure.

Parameters	Satisfactory (n = 21)	Failed (n = 13)	*p*-Value
**Age**	39.1 ± 18.8	51.8 ± 14.1	0.056
**VUDS parameters**			
**BND (n = 11)**	9 (42.8%)	2 (15.3%)	0.565
**BND + DV (n = 1** **2** **)**	7 (33.3%)	5 (38.5%)	
**NBND (n = 2)**	1 (4.7%)	1 (7.7%)	
**NBND + DSD (n = 9)**	4 (19.0%)	5 (38.5%)	
**FSF (mL)**	125.9 ± 53.4	132.7 ± 57.9	0.737
**FS (mL)**	212.2 ± 92.0	200.9 ± 79.7	0.729
**US (mL)**	266.4 ± 116.0	249.0 ± 103.2	0.674
**Compliance**	74.0 ± 63.0	94.1 ± 104.7	0.490
**Pdet (cmH_2_O)**	36.2 ± 23.4	42.5 ± 30.9	0.514
**Qmax (mL/s)**	6.8 ± 5.1	8.8 ± 6.9	0.351
**VoL (mL)**	188.3 ± 181.0	192.6 ± 166.5	0.947
**PVR (mL)**	122.2 ± 124.2	118.2 ± 152.1	0.935
**CBC (mL)**	310.5 ± 140.9	310.8 ± 144.7	0.995
**VE**	0.56 ± 0.40	0.64 ± 0.40	0.602
**BOOI**	27.7 ± 23.8	52.5 ± 30.8	0.047
**BCI**	70.3 ± 33.5	86.6 ± 35.9	0.205

Abbreviations: BND: bladder neck dysfunction, NBND: neurogenic bladder neck dysfunction, DV: dysfunctional voiding, DSD: detrusor sphincter dyssynergia, FSF: first sensation of filling, FS: fullness sensation, US: urge sensation, Pdet: detrusor pressure, Qmax: maximum flow rate, PVR: post-void residual, CBC: cystometric bladder capacity, VE: voiding efficiency, BOOI: bladder outlet obstruction index, BCI: bladder contractility index.

## Data Availability

The original contributions presented in this study are included in the article. Further inquiries can be directed to the corresponding author.
